# Decoding fractional flow reserve/instantaneous wave-free ratio discordance: is flow the answer?

**DOI:** 10.1007/s12471-023-01822-y

**Published:** 2023-10-10

**Authors:** Lokien X. van Nunen, Peter Damman

**Affiliations:** Department of Cardiology, Radboud University Medical Centre, Nijmegen, The Netherlands

That functional testing to assess the haemodynamic significance of a particular coronary artery stenosis is superior to solely visual assessment is indisputable when making decisions regarding revascularisation. In past decades, both fractional flow reserve (FFR) and coronary flow reserve (CFR) have emerged as two prominent methods to assess haemodynamic significance. CFR is defined as the ratio between maximum and resting coronary flow and is measured using intracoronary Doppler or bolus thermodilution. While CFR has proven prognostic implications [1], its use is limited by the absence of a universally accepted normal value, technical challenges in obtaining good and reproducible measurements, and its dependence on stable heart rate and blood pressure. Finally, CFR is a reflection of flow in the entire coronary circulation, including both epicardial coronary arteries and microcirculation. FFR is defined as the ratio of distal to proximal coronary pressure under conditions of maximum hyperaemia. Therefore, assessing resting flow with its variable haemodynamics is not necessary. In addition, FFR purely reflects flow limitation in the epicardial coronary artery. Besides FFR, several non-hyperaemic pressure ratios have since been introduced in an attempt to abandon the need for pharmacologically induced hyperaemia, including the instantaneous wave-free ratio (iFR). We know that discordance between FFR and iFR values occurs in up to 20% of cases [2, 3].

In this light, we read with interest the article by Stegehuis et al. published in this issue of the *Netherlands Heart Journal* [4]. We commend the authors on this deep dive into coronary physiology, attempting to further explain discordant FFR and iFR cases using different methods to assess coronary flow and stenosis resistance in a post hoc analysis of the IDEAL registry and DEFINE-FLOW study [5, 6]. The authors found that iFR and FFR were discordant in 15% (*n* = 97) of all cases. In this discordant subgroup, hyperaemic flow velocity and coronary flow reserve measurements showed iFR to be more in line with intracoronary flow evaluation by Doppler wire compared to FFR. Therefore, the authors conclude iFR should be the preferred index to guide revascularisation in the case of FFR/iFR discordance.

The results presented in this study should be interpreted with caution. The statistical analysis of the different Doppler wire measurements was performed in all patients, including the concordant positive and negative FFR/iFR groups. The main results and their statistical significance seem to be largely attributable to the differences between the concordant FFR+(abnormal)/iFR+ and FFR−(normal)/iFR− groups. Nonetheless, the results are almost identical to those in previous reports [2, 3].

Some additional considerations have to be mentioned regarding the complex subject of discordant physiological results. First is the role of the microcirculation. In the FFR−/iFR+ group, the reasoning offered for discordance is a blunted hyperaemic response, caused by old age and the higher prevalence of diabetes in this subgroup. However, both age and diabetes are also well-established risk factors for microvascular dysfunction and diminished coronary autoregulation, potentially leading to a lower hyperaemic response and increased resting flow. This is corroborated by the low CFR found in this study. These changes result in a lower maximum achievable flow down a coronary artery, and thus lower pressure gradient and higher FFR across a particular stenosis. The higher FFR reflects a lower potential gain in maximal coronary flow if that particular stenosis was treated. Earlier studies have shown that in the presence of microvascular dysfunction FFR remains accurate for detecting lesions responsible for myocardial ischaemia [7].

Regarding FFR+/iFR− discordance, the authors propose that a supra-normal hyperaemic response in coronary flow leads to the low FFR/high iFR discordance. The normal CFR (mean 2.4) corroborates the negative iFR in these cases. In the literature, this FFR+/iFR− discordance is most prevalent in left main or proximal left anterior descending coronary arteries, possibly due to a greater increase from baseline to hyperaemic flow owing to the large amount of subtended myocardium [8]. Unfortunately, we are not informed with regard to the exact location of the discordant cases in this study. Furthermore, it should be noted that the original DEFINE-FLOW included in this analysis found that, in patients with abnormal FFR and preserved CFR, there was a significantly higher rate of target vessel failure with medical therapy compared to patients with normal FFR and CFR [6].

Overall, this study presents valuable insights in coronary flow measurements in FFR/iFR concordant and discordant groups. It emphasises the role of the microcirculation and assessment of coronary flow in the functional assessment of epicardial disease. Selective measurements of microvascular function (index of microcirculatory resistance/hyperaemic microcirculatory resistance, microvascular resistance reserve, absolute flow measurements) are important to further explain if (part of) the discordance is attributable to microvascular dysfunction.

Furthermore, FFR/iFR discordance is common. We emphasise the role of location and extent of disease in the assessment of ischaemic heart disease, and its effects on epicardial and microvascular haemodynamics to best treat patients’ complaints and improve prognosis.

Until further evidence is available on clinical outcomes after iFR versus FFR-based revascularisation with discordant values, we believe this decision should be based on the patient’s anginal complaints, complexity of the revascularisation procedure versus pharmacological options, operator discretion taking the limitation of binary cut-offs into account, and the clinical setting of acute or chronic coronary syndrome.
